# Supplementary material for: Metatranscriptome Analysis of Sympatric Bee Species Identifies Bee Virus Variants and a New Virus, Andrena-Associated Bee Virus-1
Source: Viruses. 2021 Feb 12;13(2):291. doi: 10.3390/v13020291 (PMC7917660; doi:10.3390/v13020291)
Supplement: Supplementary file 1 [file viruses-13-00291-s001.zip › Flenniken_Viruses_Feb2021 SFigs S1-S14.pdf]

Supplemental Figure S1. Black queen cell virus (BQCV) Israel 2018 nucleotide alignments with BQCV reference sequence

A. Black queen cell virus nucleotide alignment BQCV (MH267693) and BQCV - Israel (MW397638), 90% identical (7,594/8,436 nt); nucleotide identities green, differences black vertical lines in BQCV - Israel 2018 seqence; qPCR product amplifed region illustrated by purple bar.

B. BQCV - Israel (MW397638), *unique* contigs (>400 nt) assembled from *A. mellifera* RNAseq reads aligned to consensus sequence. Coverage of the consensus sequence with unique contigs ranges between between 16-24x coverage, though it is important to note that sequencing depth is not illustrated in this figure, as individual contigs may represent numerous contigs. Therefore, the variations in contig sequences (denoted as black lines) are over-represented in this figure.

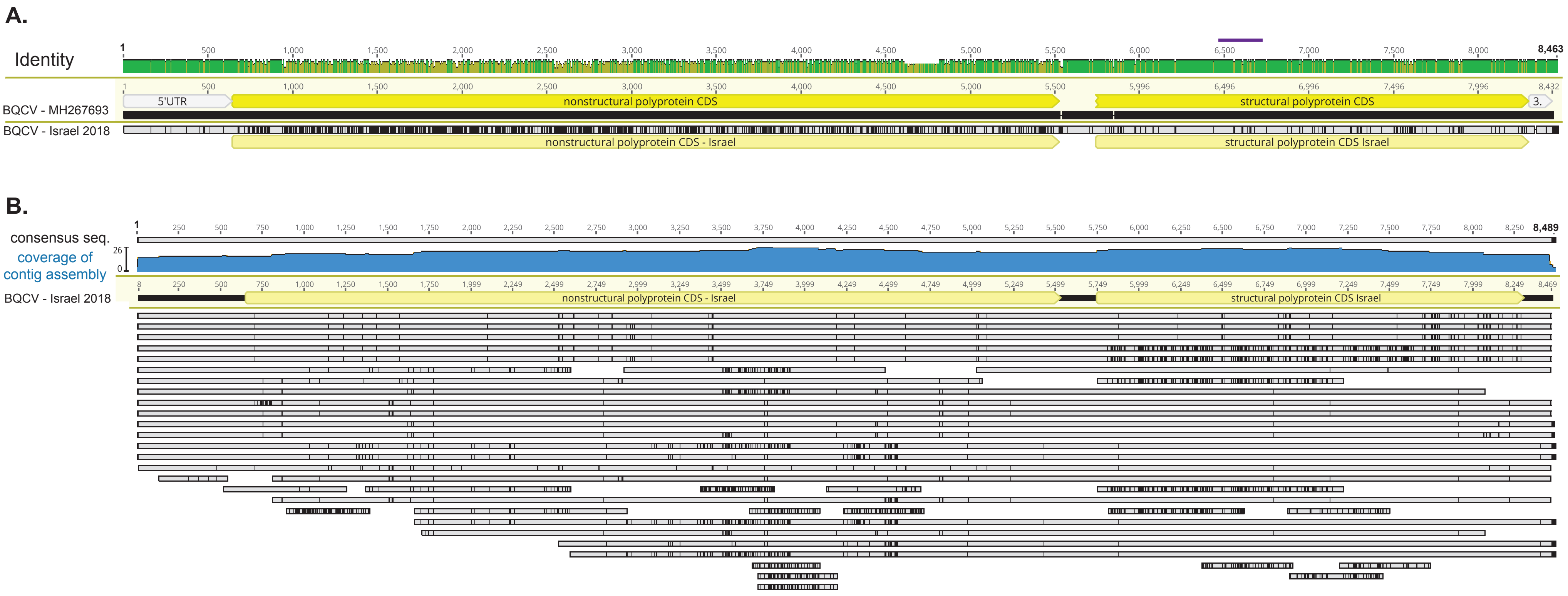

Supplemental Figure S2. Virus nucleotide alignments between reference sequence and DWV Israel 2018.

Unique contigs (> 400 nt) assembled from *A. mellifera* and *Andrena spp.* RNAseq reads aligned to DWV Israel-2018 consensus sequence (MW397639) (black bar highlighted in yellow). The overall consensus sequence of this alignment is represented by the top gray bar. Coverage of the consensus sequence with unique contigs ranges between between 17-21x coverage (blue graph), though it is important to note that sequencing depth is not illustrated in this figure, as individual unique contigs represent numerous contigs.
Therefore, the variations in contig sequences (denoted as black lines in contig sequences) are over-represented in this figure.

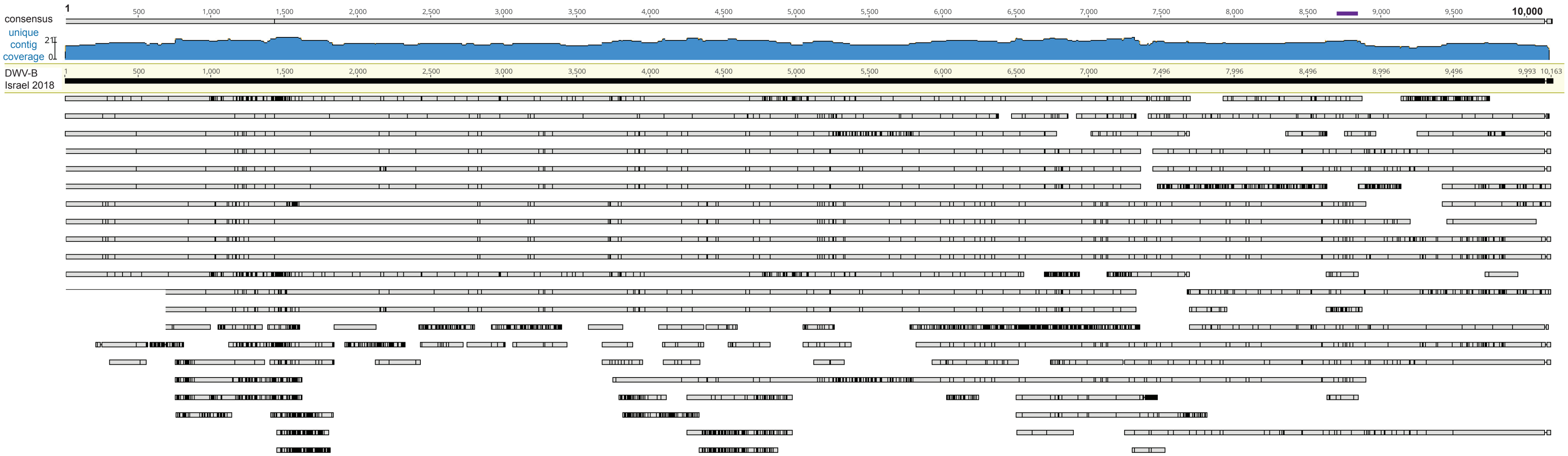

Supplemental Figure S3. Virus nucleotide alignments between reference sequences and DWV variant from Israel 2018.

A. Deformed wing virus - Israel-2018 (MW397639) best aligns with a recombinant DWV sequence (HM067438) (i.e., 9,892/10,164 nt identity, 97.3% identical), nucleotide identities are in green, differences are represented with black vertical lines; qPCR region illustrated by purple bar.
DMV-HM067438 is more similar to DWV-B/VDV-1 (HM067437) (96.2% identical) than to DWV-A (NC_004830) 90.6% identical).

B. DWV - Israel 2018 (MW397639), 5' end aligned to DWV-B genome (HM0067438), the 73 nucleotides that differ from DWV-B reference genome are denoted as colored bars in the DWV-Israel 2018 sequence (nt posion 1,160-1,540 in nt identity).

C. DWV - Israel 2018 (MW397639), 5' end (nt position 1,160-1,540 in nt identity) best aligns to DWV-A genome (NC_004830).
The 38 nucleotides that differ from DWV-A reference genome are denoted with colored bars.
The 11 nt gap in the alignment of DWV Israel 2018 and the DWV-A genome (NC_004830) is commonly found in DWV-B and DWV-A alignments.

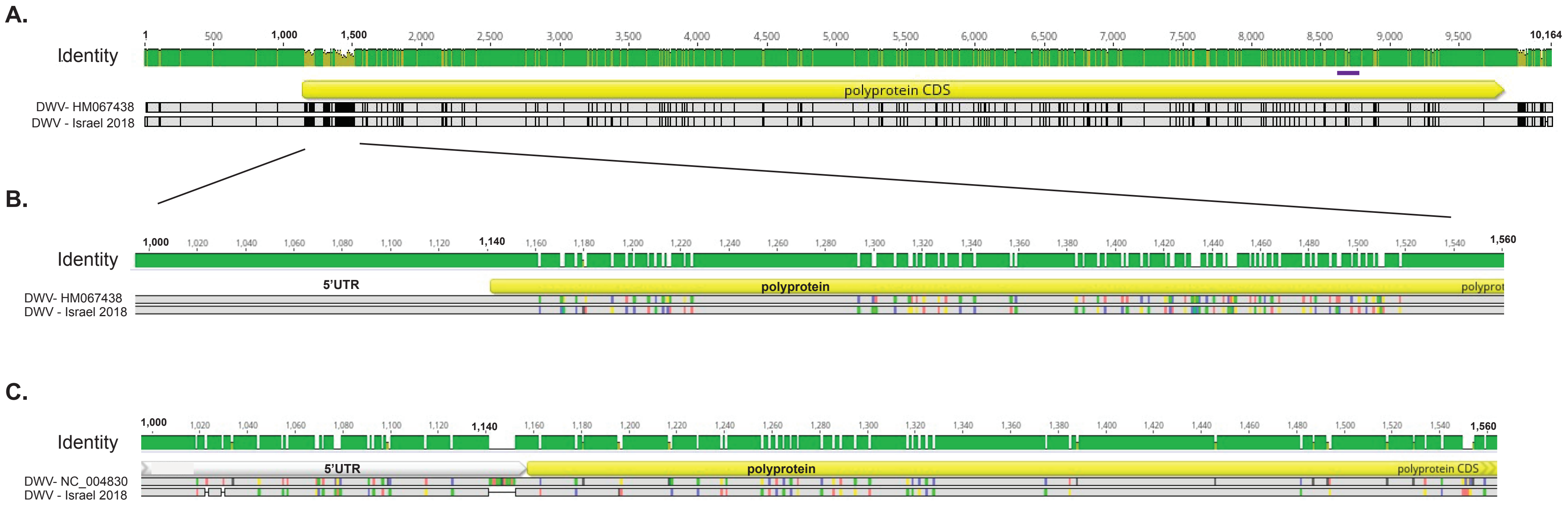

Supplemental Figure S4. Lake Sinai virus 2 Israel 2018 nucleotide alignments with LSV2 reference sequence

A. Lake Sinai virus 2 (LSV2) - Israel 2018 (MW397636) LSV2 sequence (HQ888865) (i.e., 4,552/5,937 nt identity, 76% identical), nucleotide identities green, differences black vertical lines; qPCR region illustrated by purple bar.

B. LSV2 - Israel (MW397636), *unique* contigs (> 400 nt) assembled from RNAseq reads aligned to consensus sequence illustrating up to 8x coverage of nucleotide consensus sequence with representative contigs and some variation (denoted as black lines). Sequencing depth is not illustrated in this figure, as individual contigs may represent between 2 - 49,514 transcripts per kilobase million (tpm) and between 3 - 2,404,250 reads per contig. LSV2 contig 2 (bold text) was the longest (5,997 nt) and most represented contig (i.e., 49,514 tpm and 2,403,260 estimated read counts); black lines in contigs below the reference sequence (LSV2 HQ888865, highlighted in yellow) indicate sequence variations from the consensus sequence (LSV2-Israel 2018, MW397636).

A.

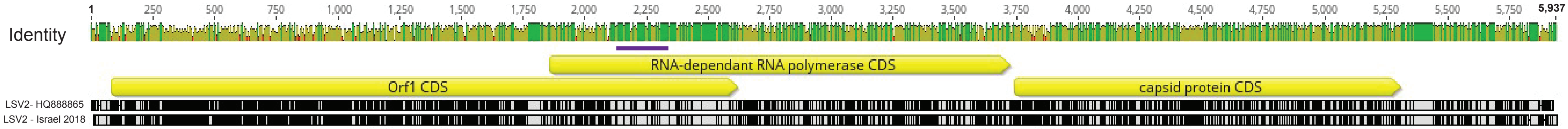

B.

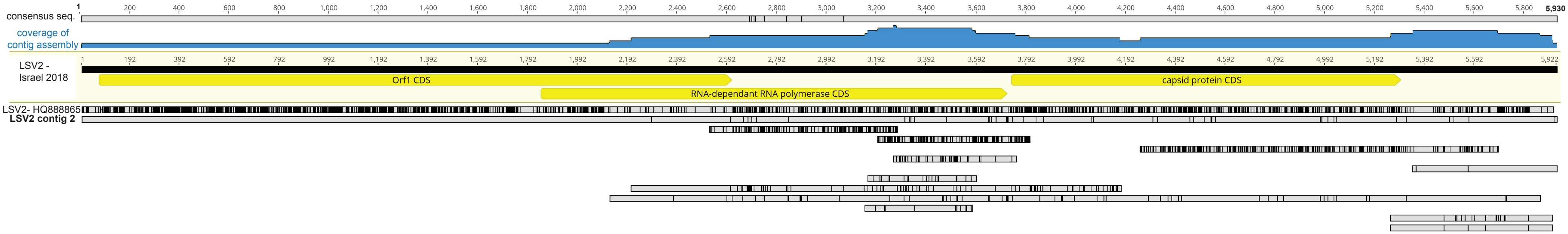

Supplemental Figure S5. Lake Sinai virus-NE Israel 2018 nucleotide alignments with LSV-NE reference sequence

A. LSV-NE Israel 2018 (MW397636) best aligns with LSV-NE sequence (NC_035113) (i.e., 5,027/5,942 nt identity, 84% identical), nucleotide identities are in green, differences represented with black vertical lines; qPCR region illustrated by purple bar.

B. LSV-NE - Israel 2018 (MW397636), *unique* contigs (> 400 nt) assembled from RNAseq reads aligned to consensus sequence illustrating up to 12x coverage of nucleotide consensus sequence with representative contigs and some variation (denoted as black lines). Sequencing depth is not illustrated in this figure, as individual contigs represent numerous contig sequences. LSV-NE contig 51 (bold text) was the longest (5,952 nt) and a well-represented contig (i.e., 9,209 tpm and 251,659 estimated read counts); black lines in contigs below the reference sequence (LSV-NE Israel-2018 MW397636, highlighted in yellow) indicate sequence variations; LSV-NE NC_035113 was included below the reference sequence for comparison.

A.

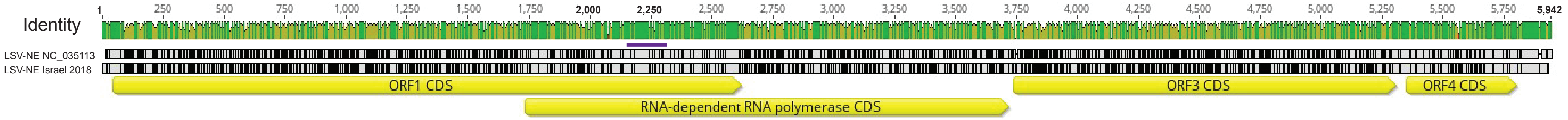

B.

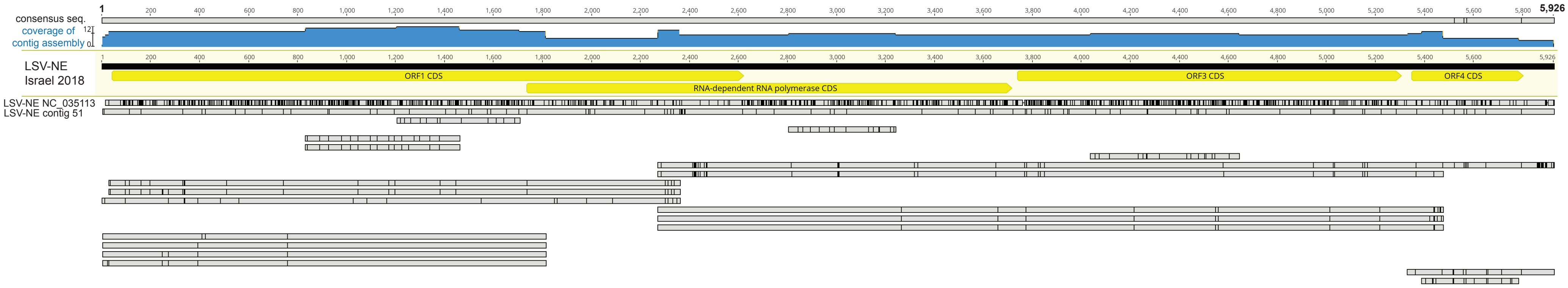

Supplemental Figure S6. Andrena associated bee virus 1 (AnBV-1) RNA 1

Andrena associated bee virus-1 (AnBV-1) RNA 1 (contig 1) is 2,005 nucleotides long and encodes four putative open reading frames (ORFs) (yellow bars). The largest, ORF1 is 1,740 nt and encodes a putative 540 amino acid (aa) protein. ORF2 of RNA1 encodes a 161 aa putative virion protein that is similar to a putative virion protein from spider-associated Loderio virus (NC_031748). AnBV-1 RNA 1 ORF2 shares 25.7% aa identity with the putative 193 aa Loderio virus virion protein.

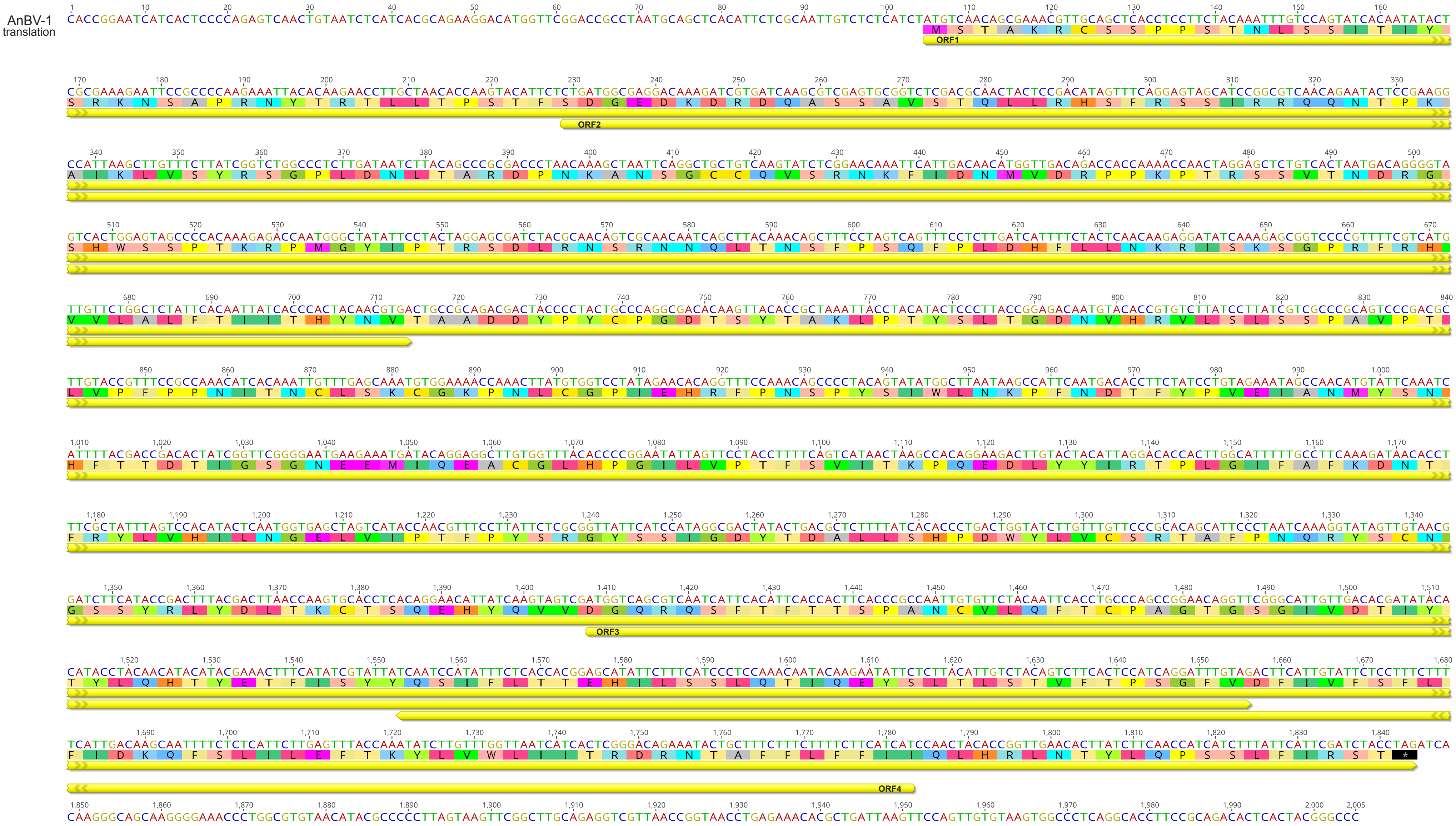

Supplemental Figure S7. Andrena associated bee virus 1 (AnBV-1) RNA 2

Andrena associated bee virus-1 (AnBV-1) RNA 2 (contig 2) with the two largest open reading frame translated . ORF 2 encodes the putative RNA-dependent RNA polymerase, amino acid position 1 - 498. Using the putative AnBV-1 RdRp amino acid sequences in a Basic local alignment sequence tool protein (BLASTp) query identifies the top hit (e-value = 0) as a putative RNA-dependent RNA-polymerase Castleton Burn virus (QAY29244) with 289/491 (59%) amino acid identity, 358/491 (72%) positive alignments, and 5/491 (1%) gaps (underlined in purple aa position aa 2 – 482).

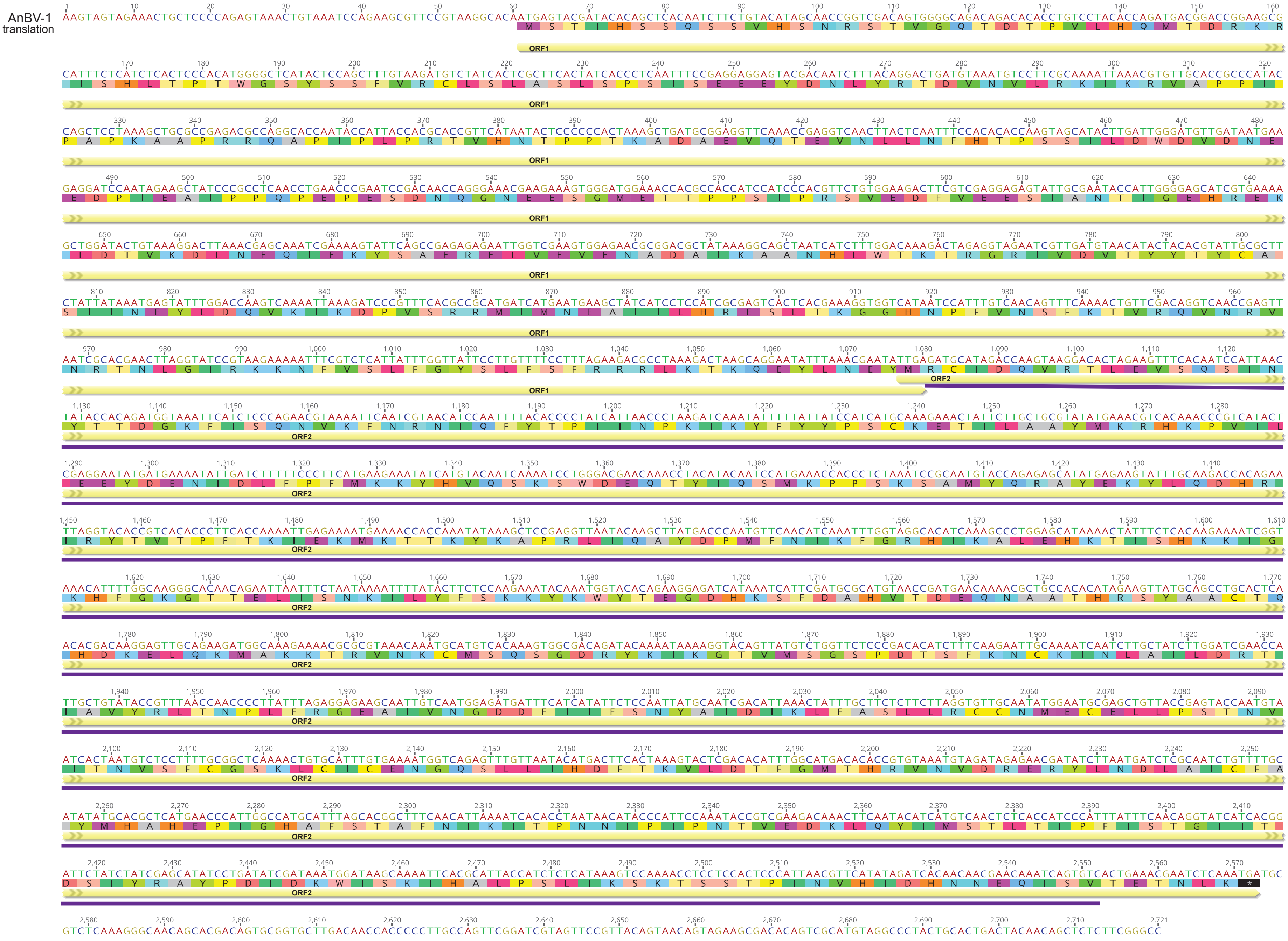

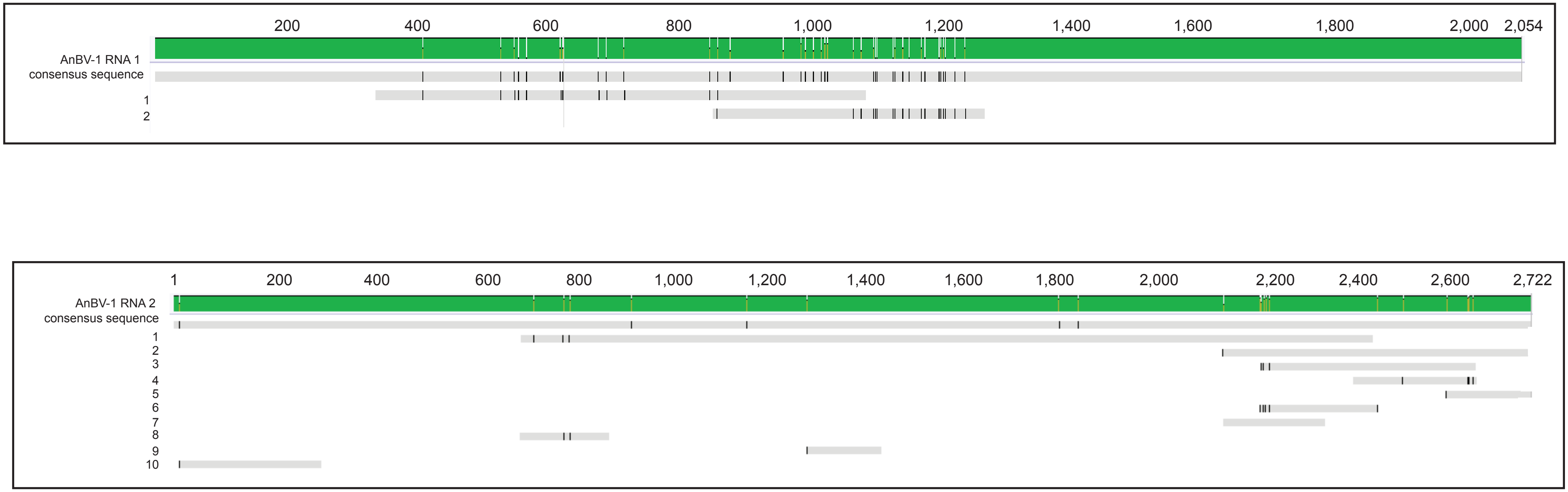

Supplemental Figure S8. Sanger sequencing confirmation of AnBV-1 RNA 1 and AnBV-1 RNA 2 sequences.
Select regions of AnBV-1 RNA 1 and AnBV-1 RNA 2 sequences were confirmed using primers designed based on the consensus sequence for PCR amplification and Sanger sequencing. Sequencing products were aligned to respective consensus sequence to confirm nucleotide identities (light gray bars).

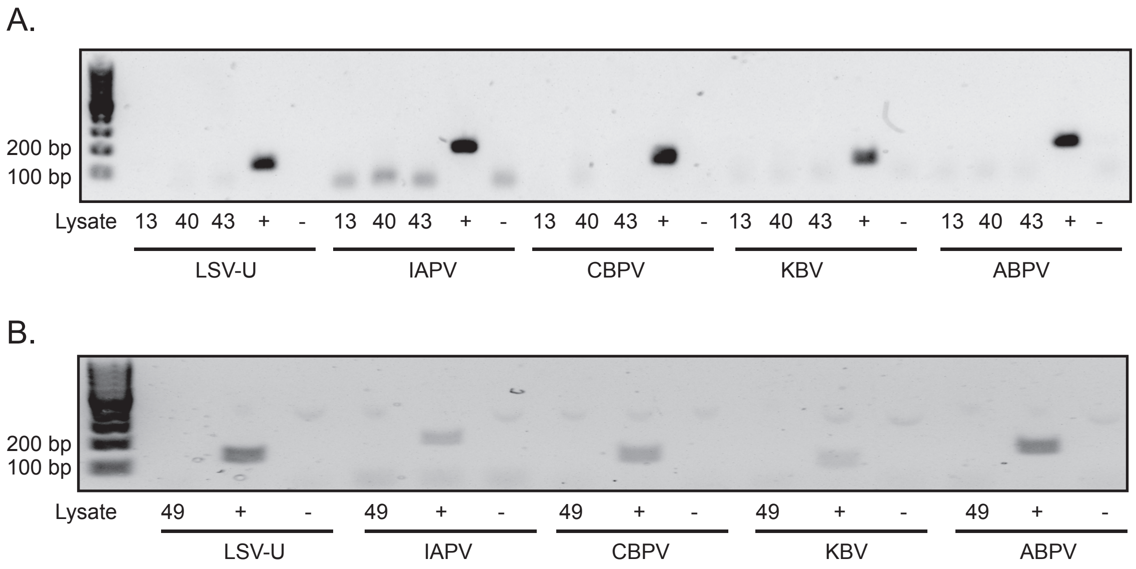

Supplemental Figure S9. Assessment of virus presence in Individual Andrena spp. lysates confirms absence of LSVs, IAPV, CBPV, KBV, and ABPV.
RNA isolated from individual AnBV-1 positive individuals (i.e., samples 13, 40, and 43, Panel A) and from an AnBV-1 negative individual (i.e., sample 49, Panel B) was reverse transcribed and used as template in PCR reactions for Lake Sinai virus - universal (LSV-U, using a primer pair that amplifies LSV1, LSV2, LSV3, and LSV4, Daughenbaugh 2015), Israeli acute paralysis virus (IAPV), chronic bee paralysis virus (CBPV), Kashmir bee virus (KBV), and acute bee paralysis virus (ABPV). Amplicons were electrophoresed through a 1.5% agarose gel and stained with SYBR Safe. The results from template-containing positive controls and template-lacking negative controls were as expected.

Supplemental Figure S10.

Andrena associated bee virus-1 (AnBV-1) RNA 1 and RNA 2 RNA-Seq coverage from virus augmented sequencing library.

Sequencing library generated from RNA that was isolated from a virus augmented (i.e., nuclease treated lysate) sample representing a single bee.

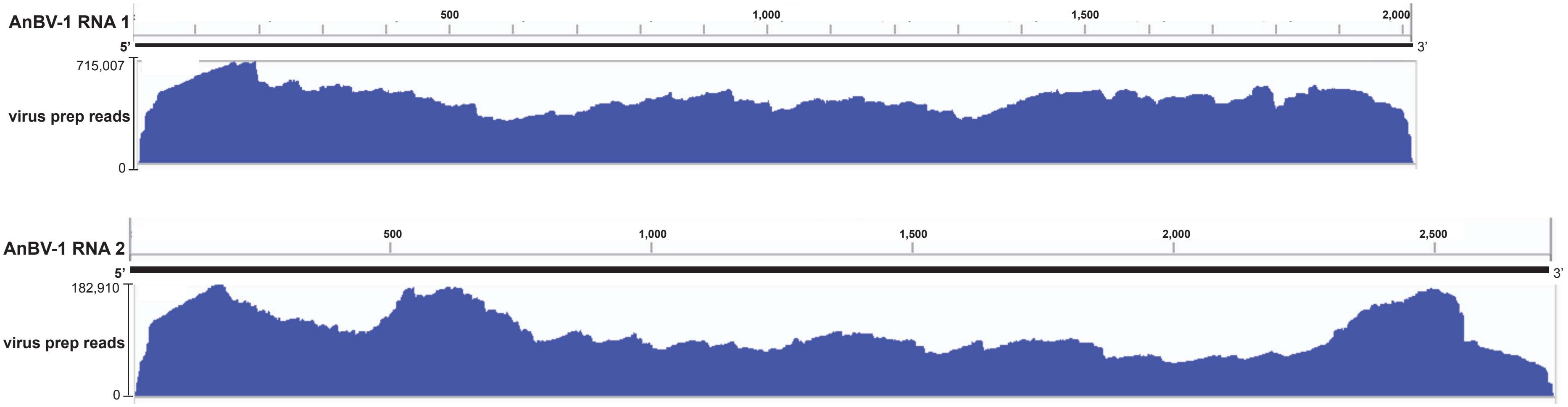

Supplemental Figure 11. Andrena associated bee virus-1 (AnBV-1) RNA 2 - RdRp

Andrena associated bee virus-1 (AnBV-1) RNA 2 encodes the putative RNA-dependent RNA polymerase, amino acid position 1 - 498 (purple). The amino acid sequences of the putative AnBV-1 RdRp amino acid sequence aligned with putative Castleton Burn virus (CBV) RdRp (QAY29244) using the Geneious alignment tool, Blosum62 with default parameters illustrates 53.3% amino acid identity (green).

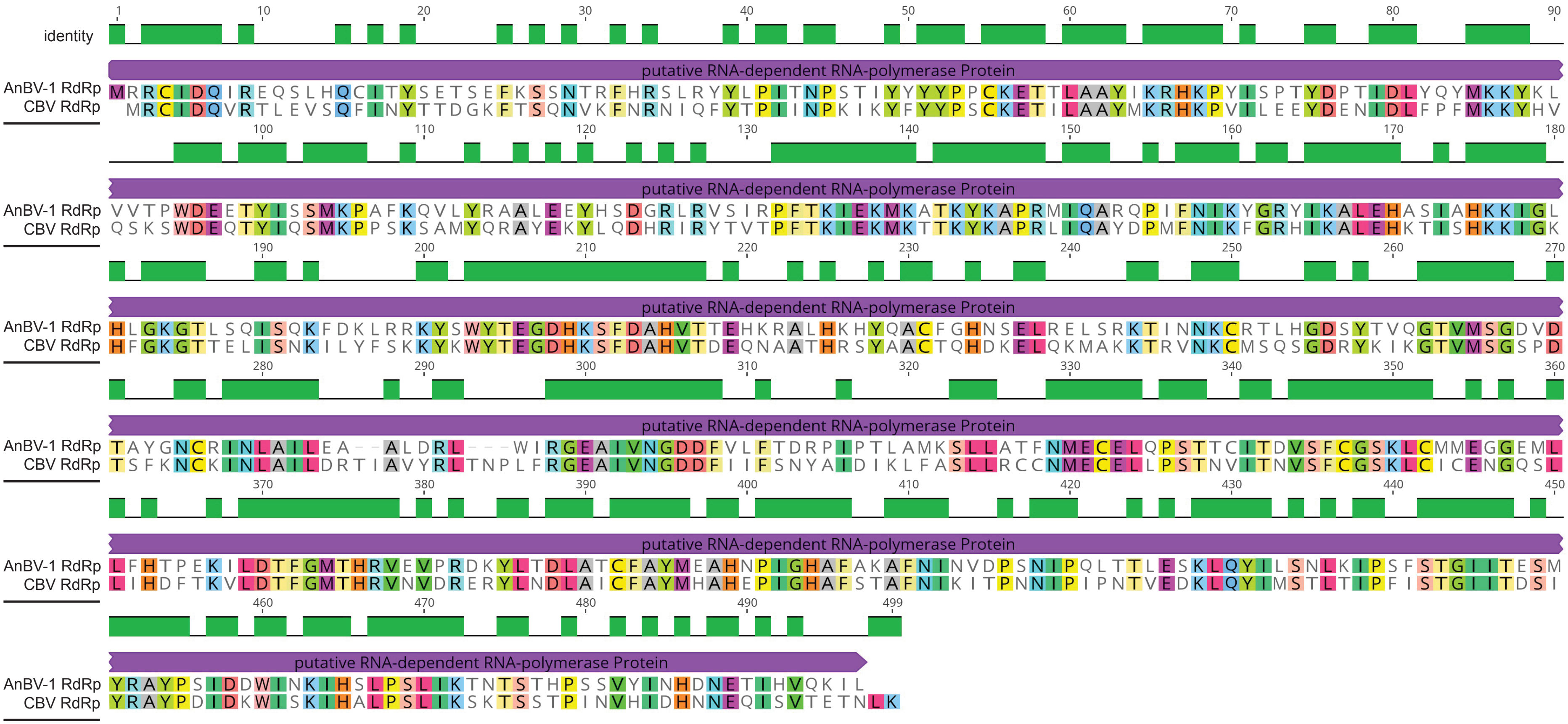

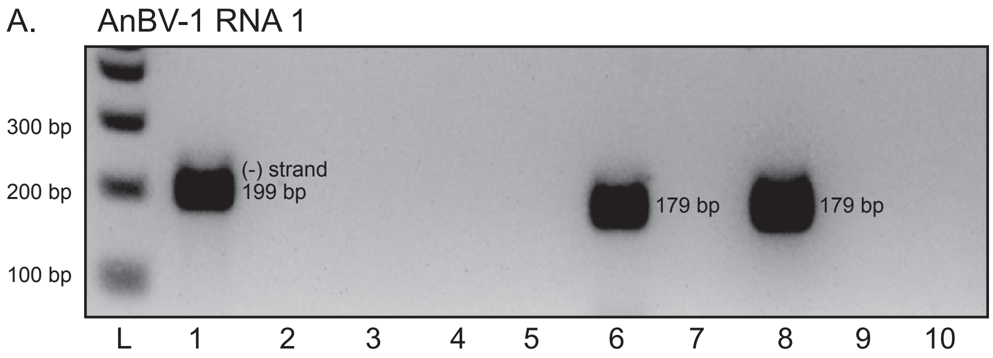

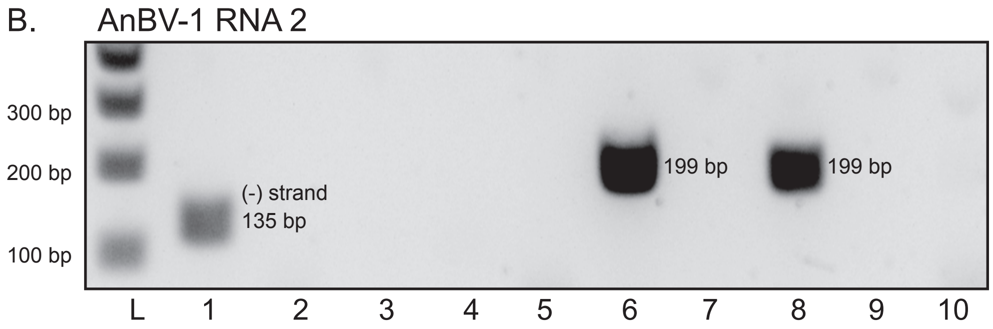

Supplemental Figure S12. AnBV-1 RNA 1 and RNA 2 negative strand detection
RNA was extracted from an AnBV-1-positive *Andrena spp.* individual and was reverse-transcribed with the primer listed below, treated with Exonuclease I to remove excess primer, and amplified using the PCR primers listed for each lane:

A. AnBV-1 RNA 1 data
L: 100 bp molecular weight ladder
1: RT with tagged negative strand-specific AnBV-1 RNA 1 primer (TAGS-AnBV-1-RNA1), PCR with TAGS and AnBV-1-RNA1-R545 primers
2: negative control: no RT in the presence of (TAGS-AnBV-1-RNA1), PCR with TAGS and AnBV-1-RNA1-R545 primers
3: negative control: RT with tagged negative strand-specific AnBV-1 RNA 1 primer (TAGS-AnBV-1-RNA1), PCR with only AnBV-1-RNA1-R545 primer
4: negative control: RT with random hexamer primer, PCR with TAGS and AnBV-1-RNA1-R545 primers
5: negative control: no RT in the presence of random hexamer primer, PCR with AnBV-1 RNA 1 PCR primers (AnBV-1-RNA1-F366 and AnBV-1-RNA1-R545)
6: positive control: RT with random hexamer primer, PCR with AnBV-1 PCR primers (AnBV-1-RNA1-F366 and AnBV-1-RNA1-R545)
7: negative control: RT with random hexamer primer, PCR with only reverse AnBV-1 RNA 1 PCR primer (AnBV-1-RNA1-R545)
8: evidence of self-priming: RT with no primer, PCR with AnBV-1 RNA 1 PCR primers (AnBV-1-RNA1-F366 and AnBV-1-RNA1-R545)
9: negative control: no template PCR with TAGS and AnBV-1-RNA1-R545 primers
10: negative control: no template PCR with AnBV-1 RNA 1 PCR primers (AnBV-1-RNA1-F366 and AnBV-1-RNA1-R545)

B. AnBV-1 RNA 2 data
L: 100 bp molecular weight ladder
1: RT with tagged negative strand-specific AnBV-1 RNA 2 primer (TAGS-AnBV-1-RNA2), PCR with TAGS and AnBV-1-RNA2-R2413 primers
2: negative control: no RT in the presence of (TAGS-AnBV-1-RNA2), PCR with TAGS and AnBV-1-RNA2-R2413 primers
3: negative control: RT with tagged negative strand-specific AnBV-1 RNA 2 primer (TAGS-AnBV-1-RNA2), PCR with only AnBV-1-RNA2-R2413 primer
4: negative control: RT with random hexamer primer, PCR with TAGS and AnBV-1-RNA2-R2413 primers
5: negative control: no RT in the presence of random hexamer primer, PCR with AnBV-1 RNA2 PCR primers (AnBV-1-RNA2-F2107 and AnBV-1-RNA2-R2306)
6: positive control: RT with random hexamer primer, PCR with AnBV-1 RNA 2 PCR primers (AnBV-1-RNA2-F2107 and AnBV-1-RNA2-R2306)
7: negative control: RT with random hexamer primer, PCR with only reverse AnBV-1 RNA 2 PCR primer (AnBV-1-RNA2-R2413)
8: evidence of self-priming: RT with no primer, PCR with AnBV-1 RNA 2 PCR primers (AnBV-1-RNA2-F2107 and AnBV-1-RNA2-R2306)
9: negative control: no template PCR with TAGS and AnBV-1-RNA2-R2413 primers
10: negative control: no template PCR with AnBV-1 RNA 2 PCR primers (AnBV-1-RNA2-F2107 and AnBV-1-RNA2-R2306)

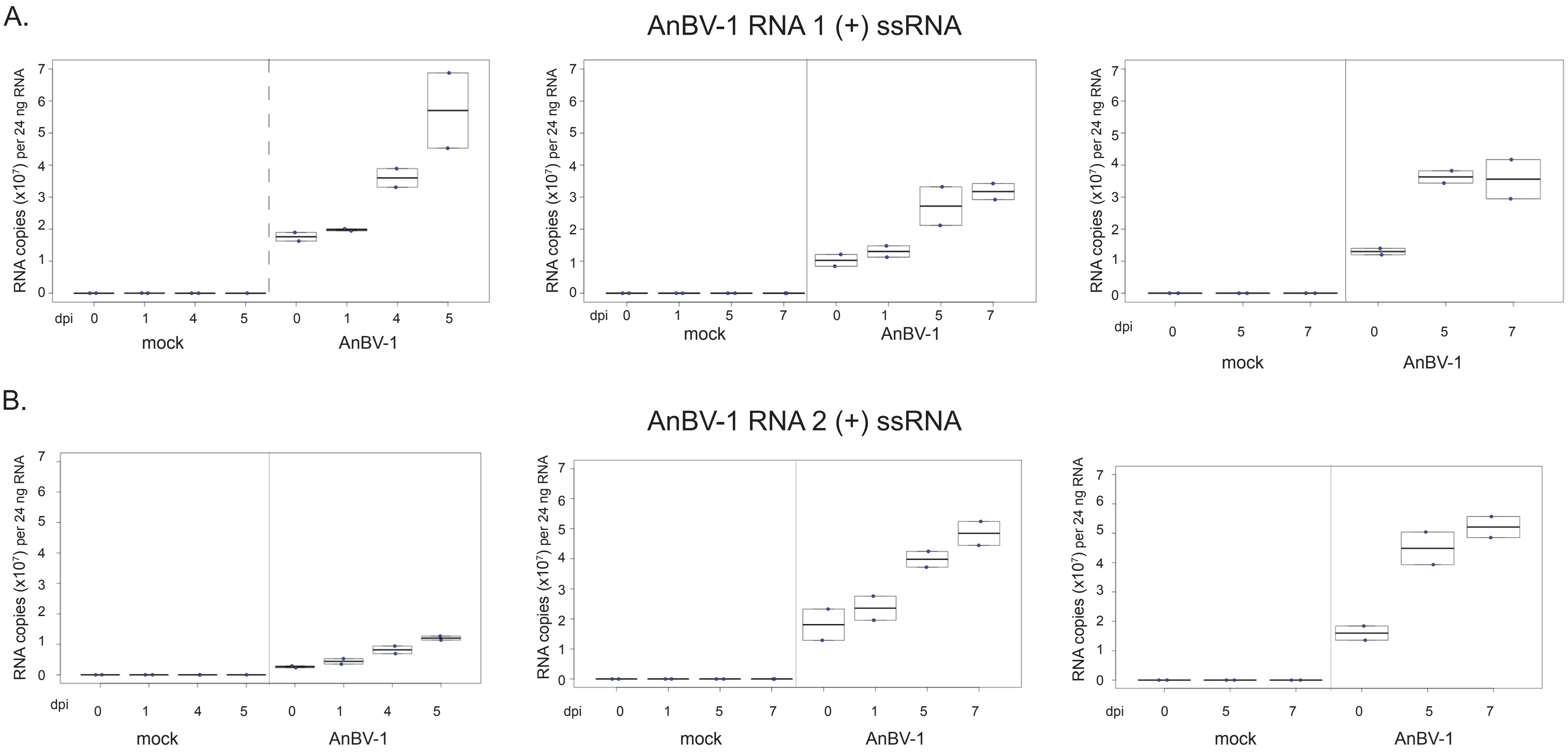

Supplemental Figure S13. AnBV-1 replicates in honey bee pupal cells - three independent replicates.
To examine the ability of AnBV-1 to replicate in primary honey bee cells, cultures of pupal cells were incubated with AnBV-1 negative lysate (mock) or AnBV-1 positive lysate (AnBV-1). Total RNA was isolated from each cell culture well at 0, 1, 4, 5, or 7 days post-infection (dpi) and virus replication was assessed via qPCR. Quantification of positive-strand, including both genome copies and transcripts, of (A) AnBV-1 RNA 1 and (B) AnBV-1 RNA 2 at each designated dpi. Relative quantity of the housekeeping gene *Amrpl8* was consistent across all samples at all time points.

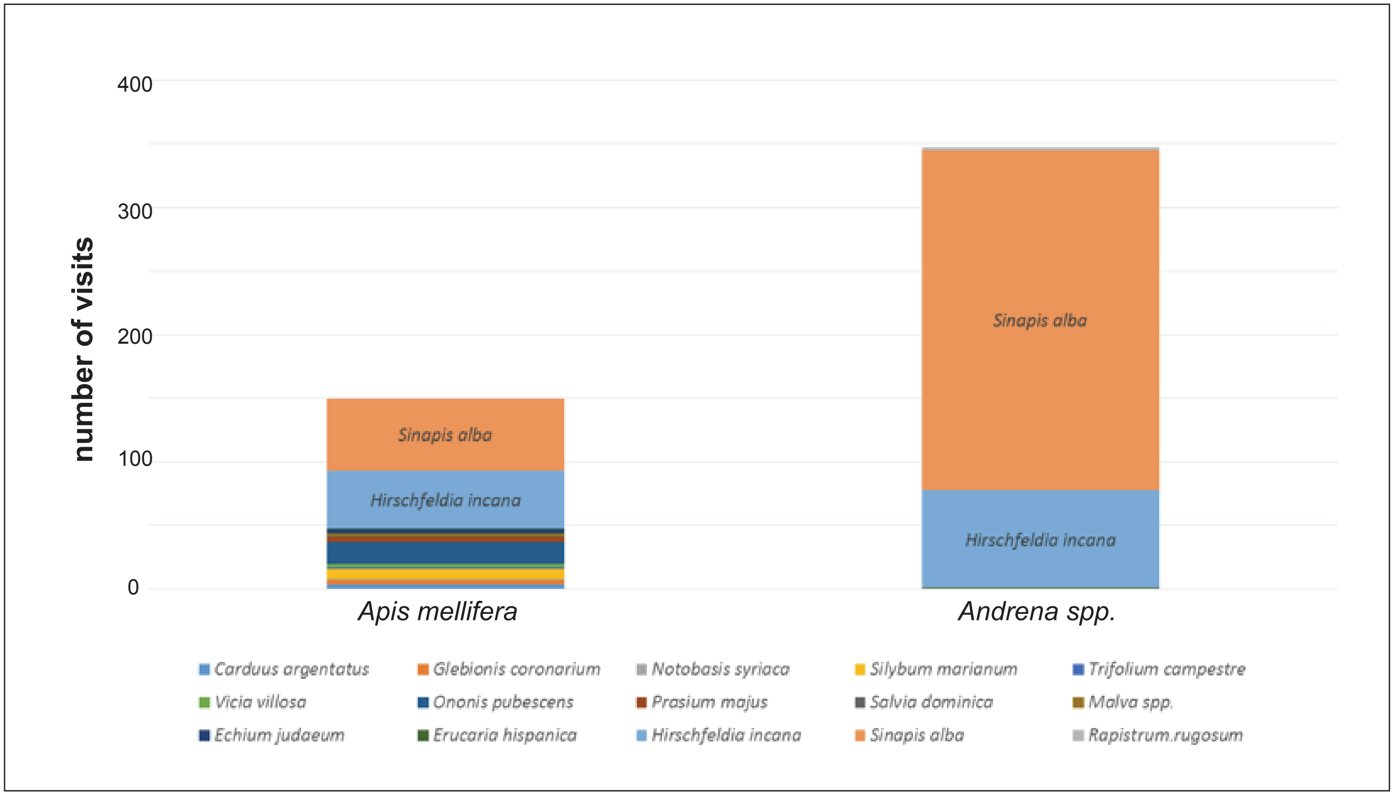

Supplemental Figure S14. Visits distribution of *Apis mellifera* and *Andrena spp.* by flower species.
Total visits by *Apis mellifera* and *Andrena spp.* (four focal species) recorded in the forager bee sample in the surveyed 14 sites, distributed by the visited flower species. The labeled flower species in the bars (*Sinapis alba* and *Hirschfeldia incana*) are the most prevalent flower species of the Brassicaceae family, dominating the flower communities in the surveyed sites.
